# Green Synthesis Characterization and Evaluation of *Senna italica* and *Vepris reflexa* Extracts and Silver Nanoparticles Against SARS-CoV-2 PLpro Enzyme

**DOI:** 10.3390/molecules31142428

**Published:** 2026-07-10

**Authors:** Rebotile M. Machika, Joshua O. Olowoyo, Malohle D. Tswaledi, Kokoette Bassey

**Affiliations:** 1 Department of Pharmaceutical Sciences, School of Pharmacy, Sefako Makgatho Health Sciences University, P.O. Box 218, Pretoria 0208, South Africa; 201609722@swave.smu.ac.za; 2 Department of Health Sciences, Marieb College of Health and Human Services, Florida Gulf Coast University, 10501 FGCU Blvd, Fort Myers, FL 33965, USA; jolowoyo@fgcu.edu; 3 Department of Biochemistry and Biotechnology, School of Science and Technology, Sefako Makgatho Health Sciences University, P.O. Box 218, Pretoria 0208, South Africa; 201012957@swave.smu.ac.za

**Keywords:** silver nanoparticles, *Vepris reflexa*, *Senna italica*, SARS-CoV-2 PLpro, phytochemicals, cytotoxicity, antiviral assay

## Abstract

This study investigated the antiviral potential of silver nanoparticles (AgNPs) synthesized from *Senna italica* and *Vepris reflexa* plant extracts, rich in saponins and flavonoids. The aim was to explore the synthesis of AgNPs through green chemistry, followed by assessing their inhibitory activity against SARS-CoV-2 papain-like protease (PLpro) and evaluating cytotoxicity. The synthesized AgNPs were predominantly spherical in shape, with an average particle size in the nanoscale range (12–55 nm), as conformed by the transmission electron microscopy (TEM) and dynamic light scattering (DLS) analyses. A dose-dependent inhibition of SARS-CoV-2 PLpro was observed, with IC_50_ values ranging from 0.12 to 0.48 mg/mL for different formulations. Cytotoxicity tests on Vero-76 cells revealed a high viability (>75%) at concentrations below 0.5 mg/mL for all AgNP samples. The findings suggest that the plant-derived AgNPs exhibit significant antiviral activity and minimal cytotoxicity, supporting their potential for further development as therapeutic agents.

## 1. Introduction

The global emergence of infectious diseases like COVID-19, caused by SARS-CoV-2, has highlighted the urgent need for effective antiviral treatments that are both effective and safe [1,2,3]. A key therapeutic target is the papain-like protease (PLpro), also known as 3CLpro, an essential viral enzyme responsible for polyprotein processing and immune invasion during SARS-CoV-2 infection, which is a validated therapeutic target in antiviral drug development [4,5]. Inhibiting PLpro activity can directly disrupt the replication cycle of the virus, making it a strategic focus for identifying potential antiviral agents [6]. However, currently available antiviral therapies remain limited, and there is a continued need for alternative antiviral strategies derived from sustainable and biologically compatible sources. Silver nanoparticles (AgNPs) have gained a lot of attention due to the broad spectrum they have in antimicrobial and antiviral properties [7,8,9]. In medicine, AgNPs are renowned for their potent antimicrobial, antiviral, and anti-inflammatory properties, making them useful in drug delivery systems, wound dressing, and coatings for medical devices [10,11]. They are also widely available and affordable, making them a practical alternative to expensive chemicals. Green synthesis of AgNPs using plant extracts has become an increasingly popular approach due to its environmental friendliness, accessibility, and biocompatibility [9,10,11,12]. Phytochemicals such as saponins and flavonoids play a crucial role in this process, contributing not only to nanoparticle formation and stabilization but also to biological activity [13,14]. Although numerous studies report the green synthesis of AgNPs using crude plant extracts, there is a lack of systematic investigations that directly compare the antiviral efficacy and cytotoxicity of specific phytochemical-rich extracts and their corresponding AgNP formulations, particularly against SARS-CoV-2 proteases. *Senna italica Mill. Subsp arachoides (Burch.) Lock* and *Vepris reflexa I. Verd* are two indigenous South African plants that are known for their high phytochemical content, including saponins and flavonoids, which possess antioxidant, anti-inflammatory and antimicrobial activities [15,16,17]. However, their potential as green reducing agents for AgNP synthesis, and the comparative antiviral performance of their saponin and flavonoid-derived nanoparticles against SARS-CoV-2 PLpro, remain largely unexplored. By eliminating the need for harsh chemicals, these natural extracts make the whole process safer and more sustainable, reflecting a growing shift towards greener, more responsible scientific practices [14].

Therefore, aim of the study was to address the research gap by synthesizing AgNPs using saponin and flavonoid (SF)-rich extracts of *S. italica* and *V. reflexa* via a green synthesis approach and to evaluate their antiviral activity against SARS-CoV-2 PLpro, focusing on harnessing the natural reducing and stabilizing agents found in these extracts. This study further sought to compare the inhibitory potential and cytotoxic profiles of the crude extracts with their corresponding AgNP formulations using Vero cell lines. Comprehensive physicochemical characterization was performed to elucidate nanoparticle size, morphology, stability, and functional group interactions. By addressing both antiviral efficacy and biocompatibility, this work provides novel insight into the therapeutic potential of plant-mediated AgNPs as sustainable antiviral candidates.

## 2. Results

### 2.1. Mass and Percentage Yields of the SAFs from S. italica and V. reflexa

The extraction of saponins and flavonoids from *S. italica* and *V. reflexa* yielded measurable quantities that confirmed the efficacy of the extraction protocols. As shown in Table 1, *V. reflexa* produced the highest yield of saponins (25.96%), followed by flavonoids (16.53%). *S. italica* recorded 10.48% and 18.58% for saponins and flavonoids respectively. These findings suggest that both species are rich in SFs, particularly *V. reflexa* in terms of saponin content, which aligns with the previous literature highlighting its phytochemical abundance [18,19].

### 2.2. UPLC-MS Analysis and Identification Marker Saponins and Flavonoids

**Figure 1:**
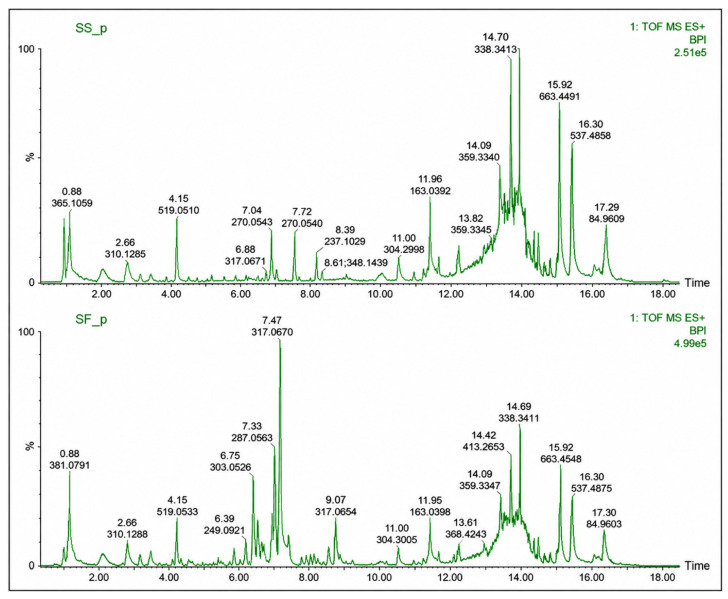
The UPLC-MS chromatogram of the *S. italica* saponins, SS (**top**) and flavonoids SF (**bottom**).

The UPLC-MS chromatograms of the *S. italica* saponins, SS (top) and flavonoids, SF (bottom) are displayed in Figure 1.

**Figure 2:**
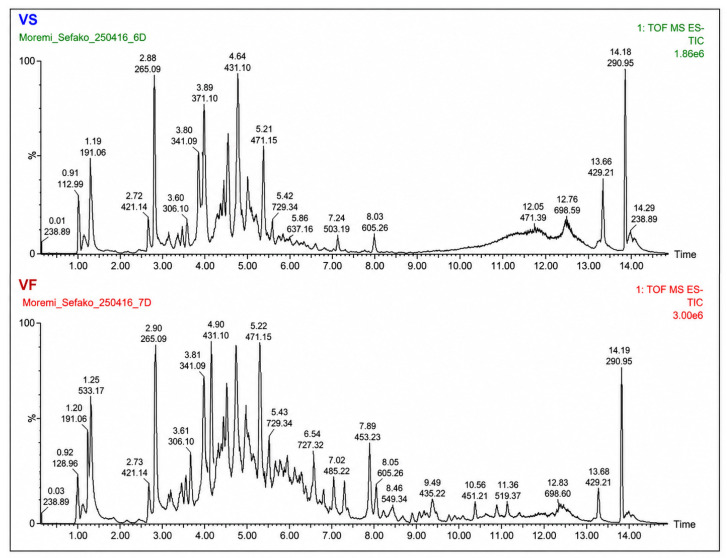
The UPLC-MS chromatogram of the *V. reflexa* saponins, SS (**top**) and flavonoids, SF (**bottom**).

The LC-MS chromatographic analysis of the saponin and flavonoid extracts from *S. italica* (Figure 1) and *V. reflexa* (Figure 2) revealed multiples peaks corresponding to various phytochemicals constituents. The identification of saponins and flavonoids (SFs) from both plant species was performed at level 2 identification confidence, based on MS/MS spectral matching with library data, supported by the retention time and literature comparison. The LC-MS data were analyzed using the LOTUS (Natural Products Occurrence Database) platform [20].

For *S. italica*, the saponin fraction showed the presence of Soyasaponin I (Rt 7.03 min) [21]. The flavonoid fraction contained compounds tentatively identified as kaempferol-3-O-rutinoside (Rt 4.15 min) [22], 6-hydroxymusizin 8-O-β-D-glucoside (Rt 5.57 min) [23], calyculatone (Rt 7.47 min) [24], and aloe-emodin (Rt 9.06 min) [25].

For *V. reflexa*, the saponin fraction included 8-demethoxy-10-O-methylhostasine (Rt 8.62 min) [26] and nkolbisine (Rt 11.01 min) [27], while the flavonoid fraction identified in [M-1] mode showed luteolin 7-O-β-D-glucoside (Rt 5.85 min) [28], ethyl ferulate (Rt 7.24 min) [29], and genistin (Rt 6.20 min) [30]. The variation in peak intensities and retention times across the samples reflects differences in phytochemical composition between the two plant species.

### 2.3. Characterization of Ag Nanoparticles

#### 2.3.1. UV-Vis Spectroscopy Analysis

**Figure 3:**
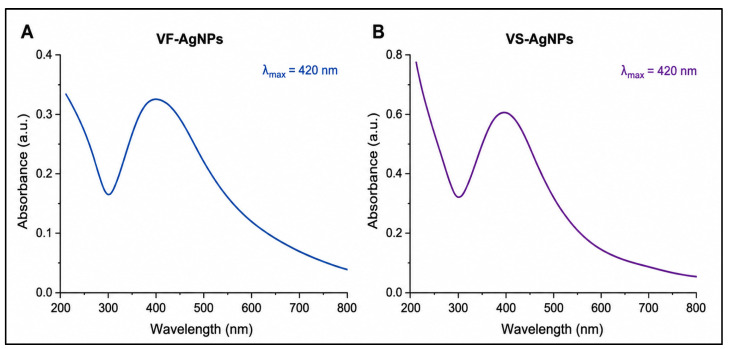
UV-vis spectra of silver nanoparticles synthesized using (**A**) flavonoid extract and (**B**) saponin extract from *Vepris reflexa*, showing characteristic surface plasmon resonance peaks, indicating successful nanoparticle formation.

**Figure 4:**
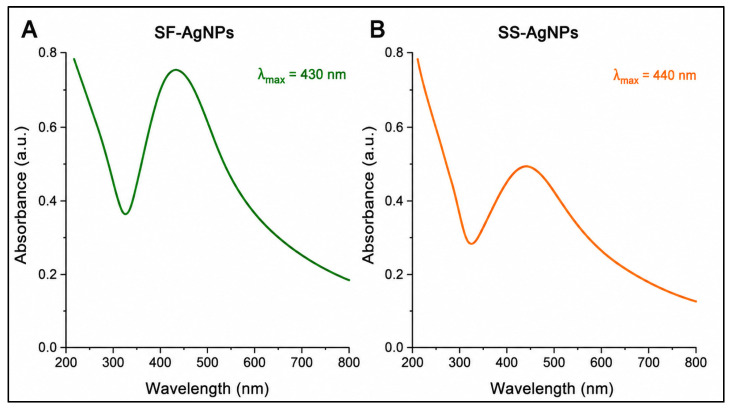
UV-vis spectra of silver nanoparticles synthesized using (**A**) flavonoid extract and (**B**) saponin extract from *Senna italica*, showing characteristic surface plasmon resonance peaks, indicating successful nanoparticle formation.

Silver nanoparticles are commonly known to display a yellowish-brown color when suspended in water, a result of surface plasmon resonance in the metal nanoparticles, typically observed in the 380 to 440 nm range (Figure 3 and Figure 4) in a silver nanoparticle solution.

#### 2.3.2. X-Ray Diffraction Pattern for Ag Nanoparticles

**Figure 5:**
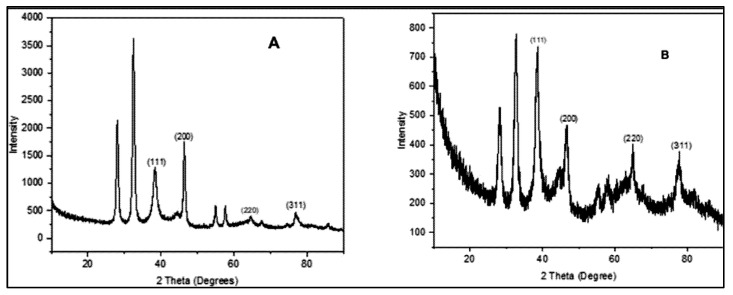
Powder X-ray diffraction (pXRD) chromatograms of SS-AgNP (**A**) and SF-AgNP (**B**) nanoparticles, confirming the crystalline structure, with peaks at (111), (200), (220), and (311).

**Figure 6:**
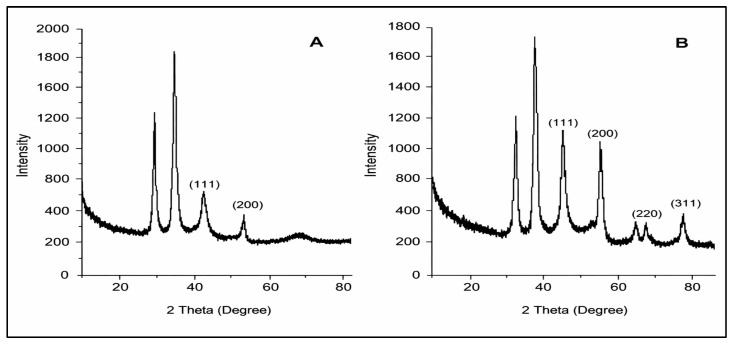
Powder X-ray diffraction (pXRD) chromatograms of VS-AgNP (**A**) and VF-AgNP (**B**) nanoparticles, showing diffraction peaks corresponding to face-centered cubic structures.

Analysis of the diffraction peak’s locations and intensities can reveal information about a sample’s crystal structure, lattice parameters, and phase composition. The XRD analysis of AgNPs synthesized using *V. reflexa* and *S. italica* extracts confirms the crystalline nature of the nanoparticles, exhibiting characteristic peaks corresponding to the face-centered cubic (FCC) structure of silver (Figure 5 and Figure 6). Diffraction peaks identified at approximately 38°, 44°, 64°, and 77° correspond to the (111), (200), (220), and (311) planes of metallic silver, respectively. Although the (111) plane at 38° is characteristic of FCC silver, it was not the most intense reflection in the analyzed samples, with stronger peaks observed at lower diffraction angles, and some patterns exhibiting more pronounced peaks at these angles. These discrepancies may be ascribed to variations in crystallite size, preferred orientation, and the effects of phytochemical capping agents present in the plant extracts.

#### 2.3.3. FT-IR Analysis of Functional Groups

**Figure 7:**
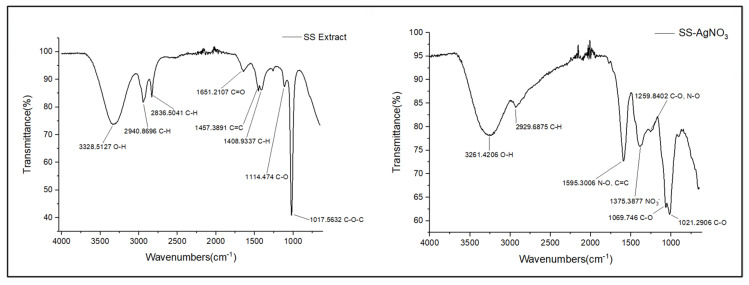
FT−IR spectrum of *S. italica* saponin extract (SS) and *S. italica* saponin silver nanoparticles (SS-AgNPs).

**Figure 8:**
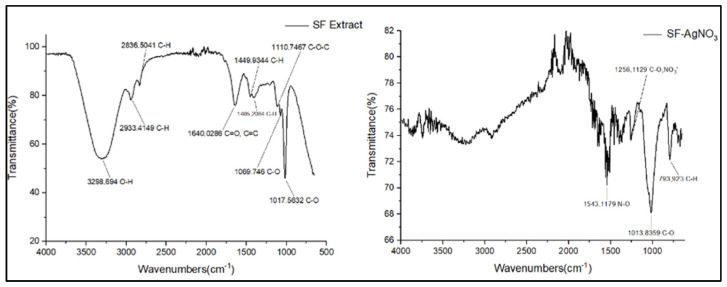
FT−IR spectrum of *S. italica* flavonoid extract (SF) and *S. italica* flavonoid silver nanoparticles (SF-AgNPs).

FTIR analysis of the four plant extracts with their corresponding synthesized silver nanoparticles provided useful insights into the functional groups involved in the nanoparticle synthesis. As shown in Figure 7 and Figure 8, all four extracts showed a broad absorption band between approximately 3284 and 3298 cm^−1^. This corresponds to O–H stretching, which is typically found in hydroxyl groups of flavonoids and saponins. These groups are not only effective in reducing silver ions to nanoparticles but are also known for their antioxidant and antiviral properties [31]. The presence of C–H stretching bands around 2835–2922 cm^−1^ indicates aliphatic chains, which are believed to help the plant compounds interact with and disrupt the viral membrane [14]. Strong absorption peaks around 1640–1703 cm^−1^ were linked to carbonyl (C=O) and aromatic ring (C=C) vibrations; these are key features in flavonoids and saponins that can interfere with viral enzymes and block virus entry into cells [3]. Other noticeable peaks in the 1373–1449 cm^−1^ region suggest C–H bending, which may support interactions with viral surface proteins. Peaks between 1013 and 1113 cm^−1^ were tied to C–O bonds and glycosidic linkages, which help make the extracts more biologically available and active [32]. After forming the silver nanoparticles, the FTIR spectra showed several changes; for instance, peaks associated with O–H and C=O either shifted or became weaker, pointing to their active involvement in the synthesis and stabilization of the silver nanoparticles [33].

**Figure 9:**
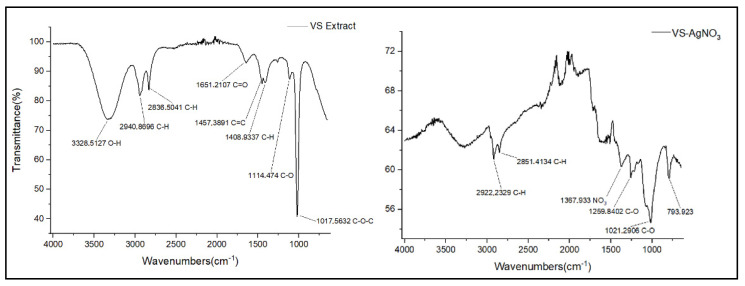
FT−IR spectrum of *V. reflexa* saponin extract (VF) and *V. reflexa* saponin silver nanoparticles (VF-AgNPs).

**Figure 10:**
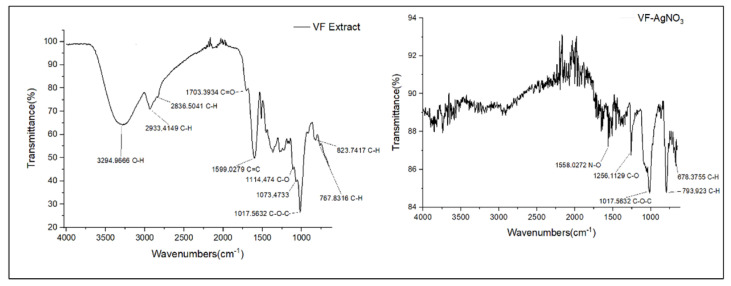
FT−IR spectrum of *V. reflexa* flavonoids extract (VF) and *V. reflexa* flavonoid silver nanoparticles (VF-AgNPs).

New peaks between 1231 and 1256 cm^−1^ and between 1543 and 1553 cm^−1^ were attributed to nitrate-related bonds, possibly from interactions with SF functional groups and the nitrate ions in silver nitrate solution (Figure 9 and Figure 10). These may help enhance the antiviral activity of the nanoparticles by improving how they interact with viruses [34]. The broad -OH stretching bands observed at 3328 and 3294 cm^−1^, characteristic of saponins, were significantly reduced after reaction with AgNO_3_, indicating the involvement of hydroxyl groups in the reduction and stabilization of silver ions. This shift confirmed the successful synthesis of silver nanoparticles [9,33], and some bands around 792–793 cm^−1^ remained visible, suggesting that parts of the plant compounds stayed attached to the nanoparticles. This helped with their stability and may also support further antiviral action.

#### 2.3.4. Particle Size Distribution by DLS

Dynamic light scattering (DLS) analysis was used to measure the size, distribution, and surface charge of silver nanoparticles (AgNPs) produced from the extracts. The results (Table 2) showed that the nanoparticles varied in size depending on the plant extract used. The smallest particles were observed in the VS-AgNP sample (35.46 nm), while the largest were in SS-AgNPs (189.9 nm). The flavonoid-based nanoparticles (SF-AgNP and VF-AgNP) generally produced smaller, more compact particles, which may be due to the stronger reducing and stabilizing capacity of flavonoids compared to saponins [10,32,35]. In terms of size distribution, the polydispersity index (PDI) values gave further insight because they help with assessing the uniformity or even sizing of the particles, with a lower PDI more favorable for the stability and quality of the nanoparticles. VS-AgNPs had the lowest PDI (0.11), suggesting a uniform particle population, while SS-AgNPs had a higher PDI (0.35), pointing to more variation in particle size and possible clustering. For biomedical uses, it is generally preferable to have a PDI below 0.3, as this indicates a more consistent and predictable formulation [36,37]. Therefore, these findings show that synthesis was successful overall, particularly for the VS-AgNPs and the flavonoid synthesized AgNPs, which showed a smaller size and uniform distribution. However, SS-AgNPs had a larger size and higher PDI, which could be because of particle aggregation.

#### 2.3.5. Zeta Potential Analysis

Zeta potential readings (Table 2), which show the stability of the nanoparticle suspension, also supported these observations. All the synthesized AgNPs had values greater than ±30 mV, a sign of strong electrostatic stability. VF-AgNPs had the highest value (+59.5 mV), while SF-AgNPs had a negative potential (−35.0 mV), possibly due to different functional groups on their surfaces. Stable nanoparticles like these are less prone to agglomeration, which is essential for a reliable performance in medical applications [11,34].

That said, while DLS offers useful information, it does have its limitations. It measures the hydrodynamic diameter, which includes not only the nanoparticle core but also the associated surface bound molecules and solvent layer in suspension. DLS is useful for assessing the particle size distribution in suspension, and in polydisperse samples, it may reveal multiple nanoparticle populations as distinct peaks in the size distribution histogram. Because of this, DLS data alone cannot give a complete picture. To obtain more accurate information about the core size and shape of the particles, we continued to use TEM which captures the actual structure of individual nanoparticles at the nanometer scale, offering much clearer and more reliable detail. Other studies also recommend combining DLS and TEM to gain a full understanding of nanoparticle characteristics [12,38].

The reported particle size and zeta potential values represent averaged measurements obtained directly from the instrument software. Future studies will include replicate measurements to provide the statistical variability of particle size and zeta potential.

The positive zeta potential values found suggest that the synthesized AgNPs had positively charged surfaces under the measurement conditions. Although plant-derived nanoparticles typically have a negative surface charge due to the adsorption of phenolic and flavonoid compounds, positive values may also occur, depending on the phytochemical composition of the extract, the adsorption of protonated biomolecules, and the physicochemical conditions of the water suspension. It is therefore possible that the surface chemistry of the current AgNPs was altered by extract-derived molecules other than negatively charged phenolics. The pH of the AgNP suspensions was not recorded during the zeta potential test, which is recognized as a restriction because pH might alter nanoparticle surface charge.

#### 2.3.6. TEM Analysis

TEM was used on dried nanoparticle powders from all four formulations (VS-AgNP, VF-AgNP, SS-AgNP, and SF-AgNP) to better understand the shape and structural uniformity of the generated silver nanoparticles (AgNPs). The TEM analysis confirmed nanoparticle morphology directly and allowed for a more precise measurement of core particle size, as DLS overestimates dimensions due to the presence of surface-bound phytochemicals and solvent layers [30].

**Figure 11:**
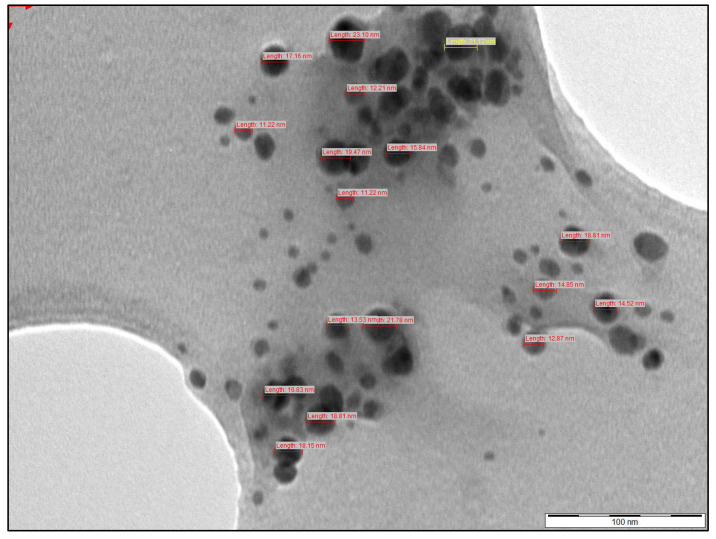
TEM micrograph of synthesized *Vepris reflexa* saponins-AgNPs (VS-AgNPs), showing predominantly spherical nanoparticle morphology.

**Figure 12:**
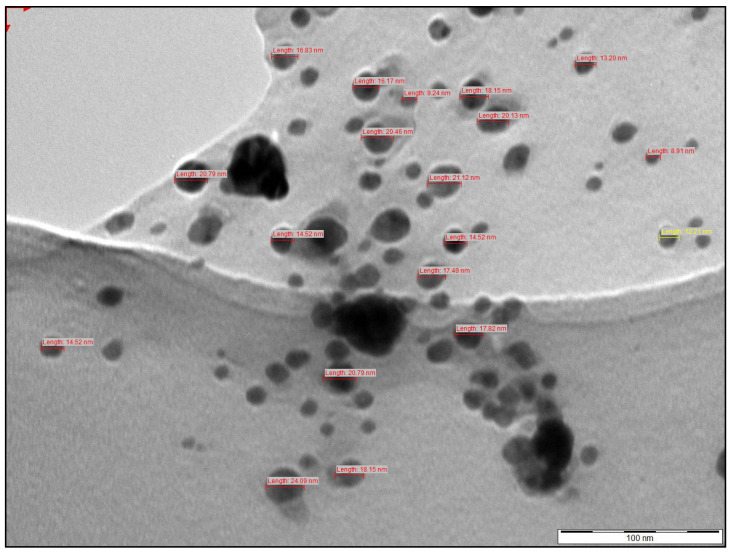
TEM micrograph of synthesized *Vepris reflexa* flavonoid-AgNPs (VF-AgNPs), showing predominantly spherical nanoparticle morphology.

**Figure 13:**
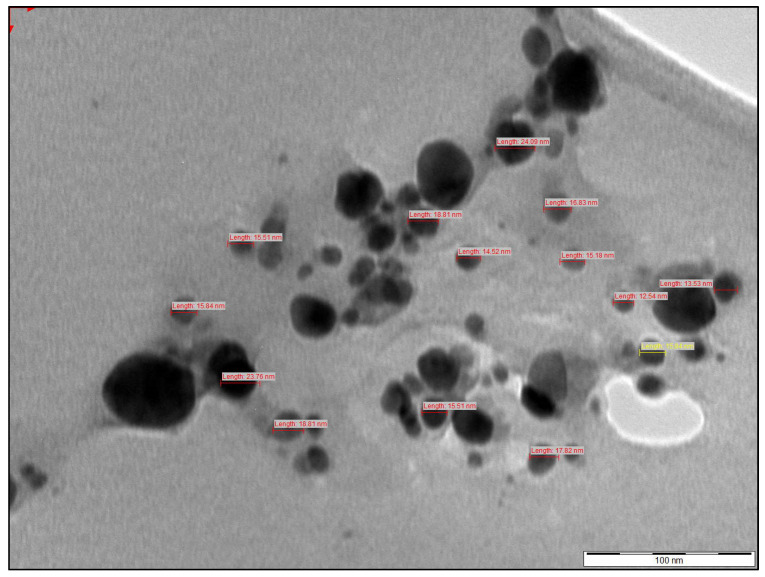
TEM micrograph of synthesized *Senna italica* saponins-AgNPs (SS-AgNPs), showing predominantly spherical nanoparticle morphology.

**Figure 14:**
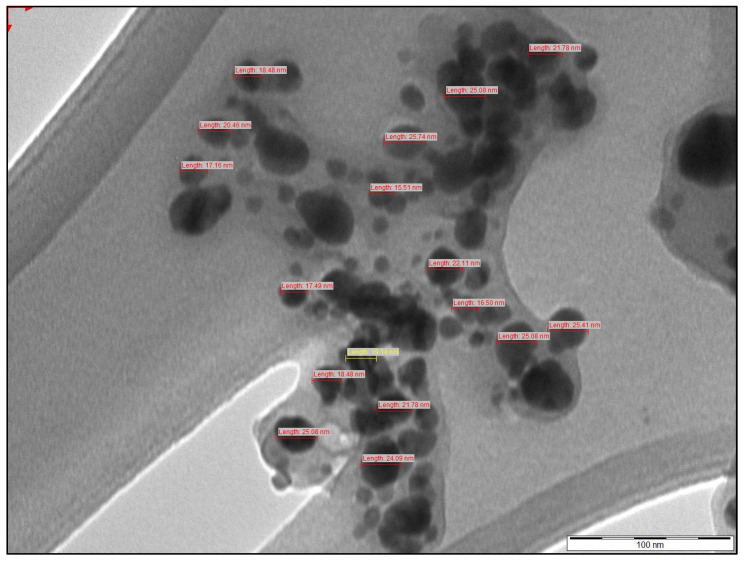
TEM micrograph of synthesized *Senna italica* flavonoid-AgNPs (SF-AgNPs), showing predominantly spherical nanoparticle morphology.

Across all samples, the particles were predominantly spherical, which showed effective synthesis. However, differences in dispersion and size uniformity were observed. VS-AgNPs (Figure 11), derived from *Vepris reflexa* saponin extract, were among the smallest (12–22 nm) and showed minimal aggregation, which correlates with their low PDI (0.11) and high zeta potential (+41.0 mV). VF-AgNPs (Figure 12) were synthesized from *V. reflexa* flavonoid extract, consistent with the DLS result (103.3 nm) and highly stable, supported by the highest zeta potential observed (+59.5 mV). On the other hand, SS-AgNPs (Figure 13), particles from *Senna italica* saponins, showed greater size variation (15–55 nm) and visible aggregation on the TEM images. This aligns with the higher PDI (0.35) and larger hydrodynamic size (189.9 nm) recorded in the DLS. In contrast, SF-AgNPs formed using *S. italica* flavonoid extracts (Figure 14) were more consistent (12–24 nm), evenly distributed, and mostly spherical. Their PDI (0.15) and negative zeta potential (−35.0 mV) confirmed strong electrostatic stabilization, likely due to the flavonoids’ abundant hydroxyl and carbonyl groups [39].

These observations emphasize the influence of the flavonoid plant extract in comparison with the saponin extract on the structural characteristics of AgNPs. Flavonoid extracts generally produced more uniform and stable nanoparticles, due to their enhanced ability to chelate silver ions and regulate nucleation kinetics [38]. The data also proved why combining DLS with TEM is important, because DLS provides valuable information on colloidal behavior in solution, while TEM gives a direct, high-resolution insight into the particle shape, dispersion, and true size, which is critical for applications in nanomedicine [40,41].

TEM micrographs confirmed the formation of predominantly spherical nanoparticles with nanoscale morphology. Due to the limited number of nanoparticles available for statistical analysis, the TEM results were interpreted qualitatively rather than quantitatively. Therefore, the TEM images were used primarily to assess nanoparticle morphology and approximate nanoscale characteristics.

### 2.4. Antiviral Inhibition of SFs and SFs-AgNPs Against SARS-CoV-2 PLpro Assay

**Figure 15:**
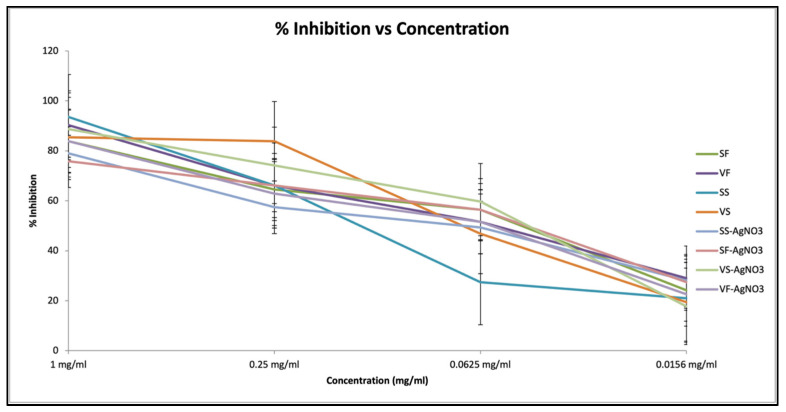
Dose-dependent inhibition of SARS-CoV-2 PLpro by SF extracts and SFs-AgNPs.

The SARS-CoV-2 papain-like protease (PLpro) is a critical viral enzyme responsible for processing viral polyproteins and modulating host immune responses. Its dual role in viral replication and immune evasion makes it an attractive target for antiviral drug development. Eight test samples, including SF extracts from *V. reflexa* and *S. italica*, were evaluated for their ability to inhibit PLpro activity using a fluorometric assay. The observed dose-dependent reductions in enzyme activity suggest that several of these extracts may contain bioactive constituents capable of interfering with the PLpro function. Positive control drugs used were the PLpro, substrate solution and inhibitor buffer, which were provided in the test kit. The percentage inhibition graph (Figure 15) provides critical insight into the efficacy of the test samples as PLpro inhibitors. Extracts demonstrated high levels of inhibition at low concentrations, with SS exceeding 90% inhibition at 1 mg/mL and retaining over 70% inhibition even at 0.0156 mg/mL. This aligns with previous studies that have demonstrated the antiviral activity of plant-derived secondary metabolites, especially flavonoids and alkaloids, against viral proteases [42]. In the context of SARS-CoV-2, AgNPs have shown potential to inhibit viral entry and replication by disrupting protease activity and viral protein stability [8]. The test of SS-AgNPs, SF-AgNPs, VS-AgNPs and VF-AgNPs afforded interesting results. From of the samples, the synthesized SS-AgNPs showed the strongest inhibition across all concentrations, surpassing 85% at the lowest dose, compared with SS with 70% inhibition at the same concentration level of 0.0156 mg/mL. These findings indicate that conjugation of SF plant extracts with AgNPs may improve their inhibitory activity against the PLpro enzyme, as reflected by the approximately 10% increase. The findings are further supported by recent research on antiviral interactions of plant extracts and metallic nanoparticles [7,43].

To improve clarity, the IC_50_ values for all the treatments are summarized in Table 3. The results showed that both plant extracts and synthesized AgNPs exhibited dose-dependent inhibitory activity against the SARS-CoV-2 PLpro enzyme. The IC_50_ values ranged approximately between 0.06 and 0.08 mg/mL, with slight variations observed among the different samples, likely reflecting differences in phytochemical composition and nanoparticle characteristics.

### 2.5. Cytotoxicity of Extracts and AgNPs on Vero Cells (MTT Assay)

**Figure 16:**
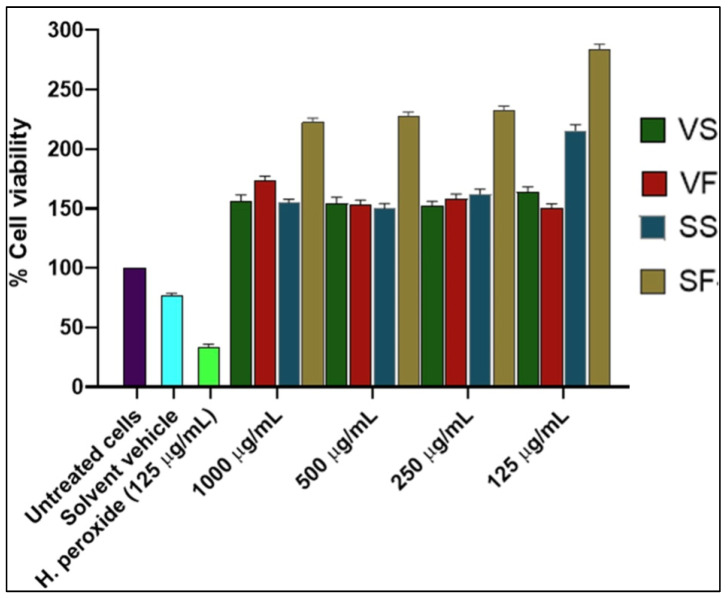
Effect of plant extracts on cell viability as determined by the MTT assay.

**Figure 17:**
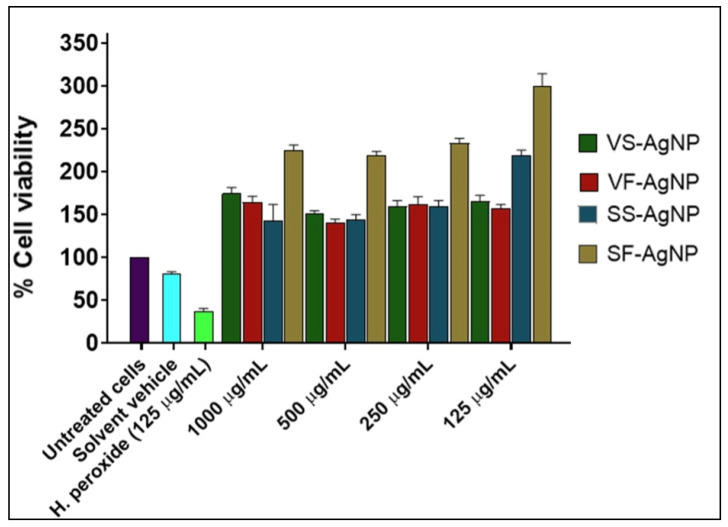
Effect of synthesized AgNPs on cell viability as determined by the MTT assay.

The cytotoxicity of the parent plant extracts (SF, SS, VF, and VS) was evaluated independently alongside their corresponding AgNP formulations, as determined using the MTT assay. The MTT assay is a reliable colorimetric method for assessing cellular metabolic activity [44,45]. This method was employed to evaluate the viability of cells exposed to varying concentrations (1 mg/mL, 0.5 mg/mL, 0.25 mg/mL, and 0.125 mg/mL) of the plant extracts and AgNPs respectively. The findings (Figure 16 and Figure 17) provided insight into the dose-dependent cytotoxic effects of both the SF crude extracts and their nanoparticle formulations, highlighting potential differences in bioactivity attributable to nanoparticle synthesis. Comparison between the two plant species and across the saponins and flavonoid extracts reveal trends relevant to therapeutic safety and efficacy.

The AgNP formulations (Figure 16) demonstrated remarkably high cell viability at all tested concentrations. SF-AgNPs exhibited the highest cell viability, increasing from 223.95% at 1.0 mg/mL to 287.39% at 0.125 mg/mL. SS-AgNPs also showed substantial viability, rising from 155.27% to 219.74%. Similarly, VS-AgNPs and VF-AgNPs, synthesized from *V. reflexa* extracts, maintained consistent viability across all concentrations. VS-AgNPs ranged from 153.45% at 1 mg/mL to 161.32% at 0.125 mg/mL, while VF-AgNPs showed 173.40% at the highest dose and 155.96% at the lowest. In contrast, the DMSO, which was used as the solvent vehicle, showed a viability range of 78.59% to 41.78%, with hydrogen peroxide serving as the positive control, showing 31.46% viability at 1.0 mg/mL.

These results suggest that the AgNP formulations did not exhibit cytotoxicity; they are more viable at activating mitosis and a tissue mutagenic effect, even at the highest concentration, supporting their potential for therapeutic applications. The conjugation of plant-derived phytochemicals to AgNPs appears to mitigate or eliminate natural cytotoxic effects associated with the free compounds, enhancing biocompatibility with mammalian cells. The results confirm that the Vero cells remained viable and metabolically active across all concentrations of the SF-AgNPs, suggesting their safety and efficacy in potential therapeutic applications [10,40,46,47,48].

Moreover, the IC_50_ values of *S. italica* extracts were approximately 0.25 mg/mL, indicating greater potency than the *V. reflexa* extracts, which had IC_50_ values above 0.5 mg/mL, although the difference was not statistically significant (*p* > 0.05). DMSO-treated cells showed reduced viability compared with untreated control cells. In addition, the effects of SF-AgNPs at 1.0 mg/mL (*p* = 0.002), 0.5 mg/mL (*p* < 0.001), and 0.25 mg/mL (*p* < 0.001) were significantly greater than those of VS-AgNPs, whereas no significant difference was observed at 0.125 mg/mL (*p* > 0.05). Furthermore, no significant difference (*p* > 0.05) was observed between the IC_50_ values of the *S. italica* extracts (0.25 mg/mL, 0.18–0.31) and their synthesized AgNPs (0.25 mg/mL, 0.15–0.32), whereas the IC_50_ values for *V. reflexa* (0.75 mg/mL, 0.68–0.83) and its derived AgNPs (0.5 mg/mL, 0.45–0.55) were significantly higher.

The cell viability results and the mean absorbance values and standard deviations for all treatments are summarized in Table 4 and Table 5. Notably, all samples exhibited cell viability values above 100% across the tested concentrations, indicating enhanced metabolic activity [49]. As a result, IC_50_ values could not be determined for these samples under the tested conditions.

The unexpectedly higher apparent cell viability recorded at 1 mg/mL for VS and VF may indicate possible interference with the MTT assay, such as direct reduction of MTT by extract constituents.

## 3. Discussion

The synthesis of silver nano-bioconjugates was carried out using leaf extracts from *V. reflexa* and *S. italica*. The formation of AgNPs was indicated by a color change from colorless to yellowish-brown color upon mixing the extracts with silver nitrate solution, which is a characteristic of surface plasmin resonance (SPR) in silver nanoparticles.

This process is attributed to the reducing capability of phytochemicals such as flavonoids and saponins, which facilitate the conversion of Ag^+^ ions to metallic silver while also acting as stabilizing agents to prevent nanoparticle aggregation, indicating the successful reduction of nanoparticles (Figure 3) and signaling the successful formation of silver nanoparticles, as shown in Figure 3 and Figure 4. UV-Vis spectra were recorded from the silver nitrate solution and the leaf extract mixture. The characteristic silver SPR band appeared at approximately 440 nm for all samples, with variation in peak intensity observed among the samples, but no shift in peak wavelength (Figure 4). The frequency and width of the surface plasmon absorption are influenced by the size and shape of the nanoparticles and the dielectric properties of both the metal and its surrounding medium. TEM images further confirmed the reduction of A^+^ to silver nanoparticles, providing visual evidence of nanoparticle formation.

The spectra for VF-AgNPs and SF-AgNPs, synthesized using flavonoid extracts, showed sharp and well-defined peaks, suggesting high crystallinity and a uniform nanoparticle size. In contrast, VS-AgNPs and SS-AgNPs, derived from saponin extracts, display slightly broader peaks, indicating larger or more polydisperse particles. The Debye–Scherrer equation could estimate the average crystallite size, with flavonoid extracts producing smaller, more uniform nanoparticles and saponin extracts yielding broader size distributions. These results demonstrate that the choice of plant extract influences nanoparticle characteristics, with flavonoid extracts being more effective at producing uniform particles.

The cytotoxicity analysis using Vero-76 cells showed that cell viability remained above 75% at concentrations below 0.5 mg/mL for all tested samples, suggesting relatively low toxicity within this concentration range. These results demonstrated that the observed antiviral activity occurred at concentrations that did not substantially compromise cell viability. The decrease in cell viability at higher doses could be attributed to a concentration-dependent cytotoxic effect, as is usual for nanoparticle-based systems due to increased cellular absorption and probable oxidative stress. However, the comparatively high survivability found at lower concentrations shows that the synthesized AgNPs have a favorable balance of biological activity and cytocompatibility. These findings support the potential use of nanoparticles as antiviral medicines, while underlining the importance of careful dose adjustment in future in vivo and clinical trials.

The inhibitory activity observed against the SARS-CoV-2 PLpro enzyme was consistent with the presence of the identified phytochemicals and the known properties of silver nanoparticles. Flavonoids such as quercetin, luteolin, and myricetin have been reported to inhibit coronavirus proteases, including PLpro, through interactions with enzyme active sites [44,50,51]. Similarly, saponins such as diosgenin derivatives have demonstrated antiviral activity in previous studies [52]. In addition, AgNPs have been shown to exhibit antiviral effects through interactions with viral proteins and interference with enzymatic processes [8,53]. The dose-dependent inhibition observed in this study aligns with these reports and suggests that both the phytochemical constituents and nanoparticle properties contribute to the observed activity. The results demonstrate that the phytochemical composition of the plant extracts plays a key role in both nanoparticle synthesis and biological activity. The combined presence of bioactive compounds and nanoscale silver structures provides a plausible basis for the observed antiviral effects, supporting the potential of plant-mediated AgNPs as candidates for further investigation against SARS-CoV-2 targets.

The high cell viability described in this study is consistent with the literature reporting that the cytotoxicity of plant-derived silver nanoparticles depends strongly on the particle size, concentration, cell type, and nature of the phytochemical capping layer [54,55]. The values observed in the presence of AgNPs may be influenced by assay interference, as nanoparticles were not removed prior to MTT addition and nanoparticles with only blanks were not included. It is known that nanoparticles and plant extracts can interact with MTT and contribute to increased absorbance values. Therefore, these results should be interpreted with caution, and further validation using alternative cytotoxicity assays is recommended. The reviews of AgNP toxicity have consistently noted that these parameters strongly influence cellular uptake, oxidative stress, and overall viability. For example, *Euryale ferox*-derived AgNPs with a mean particle size of 26.51 ± 8.87 nm showed dose-dependent cytotoxicity in Vero cells, whereas *Salacia chinensis*-derived AgNPs of about 8 nm showed no detectable cytotoxicity in fibroblasts at 100 μg/mL [56,57]. Similarly, green AgNPs synthesized from different plant extracts with sizes ranging from 16 to 20, 31 to 60, and 57 to 72 nm showed variable cytotoxicity in oral fibroblasts, further indicating that biocompatibility is formulation-dependent [58,59]. These results support the view that the synthesized nanoparticles are promising candidates for further biomedical studies, although additional toxicity validation and mechanistic investigations remain necessary before definitive therapeutic claims can be made.

## 4. Materials and Methods

### 4.1. Equipment

The synthesis and characterization of silver nanoparticles (AgNPs) from *S. italica* and *V. reflexa* required the use of various instruments. A precision weighing balance (RADWAG, Rasom, Poland) was used to measure the crude extracts and silver nitrate. A hot plate with a magnetic stirrer (LASEC, Cape Town, South Africa) ensured uniform mixing, while aluminum foil (Wyda Easy, Johannesburg, South Africa) was used to protect light-sensitive reactions. Nanoparticles were separated using a centrifuge (NUVE NF 800, Ankara, Turkey), and a freeze dryer (LABCONCO, Kansas City, MO, USA) was used for solvent removal and sample preservation. The formation of AgNPs was confirmed using UV–visible spectrophotometry (Agilent Cary 60, Santa Clara, CA, USA). Particle size and stability were analyzed using dynamic light scattering (DLS) (Microtrac Nanotract Wave II, Osaka, Japan). Functional group identification was performed using Fourier transform infrared spectroscopy (FTIR) (Agilent Cary 630, Santa Clara, CA, USA). The morphology and size of nanoparticles were examined using TEM (JEOL JEM-2100, Japan), and crystallinity was confirmed via X-ray diffraction (XRD) (Bruker D2 Phaser, Karlsruhe, ND, USA).

### 4.2. Sample Collection and Preparation

Leaves of *Senna italica Mill. Subsp arachoides* and *Vepris reflexa I. Verd* were collected from Ga-Phaahla village, Sekhukhune district, Limpopo, South Africa. Coordinates were recorded as (24°41′39.0″ S, 29°44′13.7″ E) and (24°18′0″ S, 29°38′0″ E). Plant identification was confirmed by the South African National Biodiversity Institute (SANBI), and voucher specimens were deposited at the Department of Pharmaceutical Sciences, Sefako Makgatho Health Sciences University. The collected leaves were washed, air-dried at room temperature, and pulverized into a fine powder using a lab grinder (Powteq HM100CE, Beijing, China). The powder was stored in airtight containers in a cool, dry place.

### 4.3. Extraction of Saponins and Flavonoids

Crude saponins and flavonoids (SFs) were extracted from *V. reflexa* (316 g) and *S. italica* (159 g). For saponin extraction, plant powders were heated with 20% ethanol for 4 h at 55 °C. The resulting mixture was filtered using Whatman No. 1 filter paper to remove plant debris and re-extracted. The combined extracts were concentrated under reduced pressure using a rotary evaporator to approximately 200 mL. The concentrated extract was then washed sequentially with diethyl ether and n-butanol, followed by treatment with 5% NaCl solution. The resulting saponin-rich fraction was air-dried in a fume hood at room temperature to obtain the dry crude extract. No chromatographic purification or fractionation step, including column chromatography, was performed, and the crude extract was used directly for subsequent analyses.

For flavonoids, a modified ultrasonic-assisted extraction was performed using 52% ethanol (1000 mL for *V. reflexa*, 500 mL for *S. italica*) at 30 °C for 20 min using an ultrasonic bath (KQ3200DE, Kunshan, China). The extracts were then centrifuged using a TGL-16 G centrifuge (Shanghai Anting, Shanghai, China), and the supernatants were dried. For further analyses, the dried extracts were reconstituted in methanol to a final concentration of 0.5 mg/mL, followed by sonication for 10 min to ensure homogeneity.

### 4.4. UPLC-MS Analysis of the SFSs and Identification Marker Compounds

The crude saponin extracts were dissolved in HPLC-grade acetonitrile, while the crude flavonoid extracts were dissolved in HPLC-grade acetonitrile. Each solution was vortexed and centrifuged at 4 °C and 14,000 rpm for 15 min before LC-MS analysis. The mixture was then vortexed and centrifuged at 4 °C and 14,000 rpm for 15 min. Silver nanoparticles were synthesized separately, corresponding to the saponin and flavonoid extracts of *S. italica* and *V. reflexa* respectively. After filtering, the plant SF extract was then directly injected into each of the Waters^®^ Synapt G2 high-definition mass spectrometry (HDMS) LC-MS systems for analysis. The separation and other conditions are summarized in Table 6.

### 4.5. Synthesis of Silver Nanoparticles (AgNPs)

Green synthesis of silver nanoparticles was performed by separately reconstituting the previously obtained crude saponin or flavonoid extracts in water and mixing 100 mL of each extract solution with 100 mL of 1 mM AgNO_3_ in a 250 mL amber volumetric flask. No mixed-plant extract was used. The reaction mixture was covered and stirred on a hotplate (LASEC, Cape Town, South Africa) at 40 °C for 60 min at 500 rpm. Four distinct nanoparticle formulations were synthesized independently from the individual extracts, namely SS-AgNPs (*S. italica* saponin), SF-AgNPs (*S. italica* flavonoid), VS-AgNPs (*V. reflexa* saponin), and VF-AgNPs (*V. reflexa* flavonoid). The synthesized nanoparticles were isolated by two-step centrifugation (NUVE NF 800, Turkey) at 10,000 rpm for 25 and 30 min, washed with Milli-Q water, and freeze-dried (LABCONCO, USA). The resulting AgNPs were stored in foil-wrapped Falcon tubes and kept in the dark until further characterization.

### 4.6. Characterization of Silver Nanoparticles

#### 4.6.1. UV-Visible Spectroscopy

The synthesized silver nanoparticles (AgNPs) were monitored visually and confirmed by scanning absorbance spectra (Agilent Cary 60, USA) from 250 to 800 nm, with characteristic surface plasmon resonance (SPR) peaks around 420–430 nm.

#### 4.6.2. X-Ray Diffraction (XRD)

The crystalline structure of Ag nanoparticles was confirmed using an X-ray diffractometer (Bruker D2 Phaser, USA). The sample was converted to powder and placed in X-ray diffraction cubes. The particle size was determined using Scherrer’s equation, where λ is the X-ray wavelength, B is the broadening of the diffraction line, and θ is Bragg’s law.

#### 4.6.3. Dynamic Light Scattering (DLS)

DLS measurements were performed using a NanoTrac Wave II particle size analyzer (Microtrac, Montgomeryville, PA, USA). Powdered silver nanoparticles were dispersed in Milli-Q water and centrifuged for 3 min to break down the particles prior to analysis. A small aliquot of the suspension was then transferred into the instrument sample cell using a Pasteur pipette. The instrument performed three consecutive runs per measurement at 25 °C. The NanoTrac Wave II uses a 780 nm, 3 mW laser, and it determines particle size using a backscattered laser-amplified scattering reference method with FFT power spectrum analysis. The measured values are reported as the hydrodynamic particle diameter.

#### 4.6.4. Fourier Transform Infrared Spectroscopy (FTIR)

For FTIR spectrum (Agilent Cary 630, USA) analysis, silver nanoparticle samples were centrifuged multiple times to remove impurities and concentrate the particles. The purified AgNPs were redispersed in distilled water and analyzed using FTIR to identify the functional groups responsible for reducing and stabilizing the nanoparticles. Spectra from both crude plant extracts and synthesized AgNPs were compared to determine which biomolecules, such as saponins or flavonoids, contributed to nanoparticle formation. FT-IR spectra were recorded by measuring the pallet on the laser dot in the range of 4000–650 cm^−1^ using 128 sample scans and 16 background scans at a spectral resolution of 8 cm^−1^. For improved visual presentation, the FT-IR spectra were redrawn using OriginLab 10.25 software.

#### 4.6.5. Transmission Electron Microscopy (TEM)

Transmission electron microscopy (JEOL JEM-2100, Japan) was used to investigate the morphology, distribution, and size of the produced SF-AgNPs. A drop of the evenly distributed nanoparticle suspension was deposited on a carbon-coated copper grid and left to cure at room temperature. The samples were next investigated using a TEM (JEOL JEM-2100, Akishima, Japan) with an accelerating voltage of 200 kV.

### 4.7. Cell Cytotoxicity Assay (MTT Assay)

The cytotoxicity of the plant extracts and synthesized silver nanoparticles was assessed using the MTT assay on Vero cells. The Vero cell line (ATCC^®^ CRL-1587™) was purchased from ATCC (American Type Culture Collection (ATCC, Manassas, VA, USA)), and cells were maintained and cultured in a Dulbecco’s Modified Eagle Medium (DMEM) supplemented with 1 × PSN and 10% fetal bovine serum (FBS) in a T75 culture flask at 37 °C, in a 95% humidified environment and 5% CO_2_. Vero-76 cells were seeded into 96-well plates at a density of 1 × 10^4^ cells/well and allowed to adhere before treatment with the test samples. Cell counting was performed using Cell Countess (Thermofisher, Carlsbad, CA USA), at a 1:1 cell to trypan-blue dye ratio.

Cells were seeded in 96-well plates and incubated to allow attachment, followed by treatment with various sample concentrations (0.0195–2.5 mg/mL) for 24 h. Untreated wells served as negative controls, while DMSO was used as a solvent vehicle and H_2_O_2_ was used as positive controls. After incubation, MTT solution was added, and the metabolically active cells reduced it to formazan crystals, which were later dissolved in acidic isopropanol. Absorbance was measured at 560 nm, and cell viability was calculated relative to the untreated control. Absorbance was measured at 560 nm using a microplate reader (e.g., Promega GloMax). The absorbance values directly reflected the number of viable cells. Cell viability was expressed as a percentage of the untreated control group and calculated using the formula described by [49]. Cell viability was calculated by normalizing absorbance values to untreated control cells, which were set at 100%. The MTT assay was performed without removal of nanoparticles prior to the addition of MTT reagent. Nanoparticle-only wells (without cells) were not included as blanks to correct for potential interference. Therefore, possible interaction between nanoparticles and the MTT reagent cannot be excluded. Results are presented as bar graphs with error bars indicating standard deviation. These graphs illustrate dose-dependent responses and allow for comparison across different extracts, AgNPs, and control treatments. Future validation will be performed using complementary assays, including gene expression, flow cytometry-base viability assays, and other cytotoxicity methods.

### 4.8. SARS-CoV-2 PLpro Inhibition Assay

The inhibitory activity of the SAF extracts and synthesized silver nanoparticles against the SARS-CoV-2 papain-like protease (PLpro) was evaluated using a fluorescence-based enzymatic assay, following the protocol adapted from BPS Bioscience and similar methods described by [60]. A total of 10 test samples were evaluated, which included Samples 1 to 6, consisting of SAF extracts derived from *V. reflexa* and *S. italica*. These included isolated saponins, alkaloids, and flavonoids obtained through phytochemical screening. Samples 7 to 10 consisted of silver nanoparticles synthesized using AgNO_3_ and the aqueous fractions of the above plants, namely, VS-AgNPs, VF-AgNPs SS-AgNPs, and SF-AgNPs. These were prepared using a green synthesis approach. The assay was performed in a black 96-well microplate to minimize background fluorescence and eliminate inter-well signal interference and sensitivity. Briefly, 30 µL of PLpro enzyme (0.3–0.5 ng/µL) diluted in assay buffer containing 1 mM dithiothreitol (DTT) was added to the wells designated for the positive control, inhibitor control, and test samples. Blank wells received 30 µL of assay buffer with DTT. Ten microliters (10 µL) of GRL0617 inhibitor solution (500 µM) was added to the inhibitor control wells. For the test wells, 10 µL of each sample (either extract or AgNPs) was added. To the blank and positive control wells, 10 µL of inhibitor buffer or DMSO (≤5%) was added to match final conditions. The reaction mixtures were preincubated at 37 °C for 30 min. To initiate the reaction, 10 µL of PLpro fluorogenic substrate (diluted to a final concentration of 25 µM) was added to each well. The total volume per well was 50 µL. Plates were sealed and incubated at 37 °C for 45–60 min. Fluorescence was measured using a microplate reader at an excitation wavelength of 360 nm and emission at 460 nm. After 12 h of incubation, the fluorescence was measured again.

## 5. Conclusions

This study demonstrated that saponin and flavonoid-rich extracts of *S. italica* and *V. reflexa* can be effectively used for the green synthesis of silver nanoparticles with consistent physicochemical characteristics. The synthesized AgNPs exhibited nanoscale particle sizes, acceptable polydispersity, and stable surface charge, confirming successful formation and colloidal stability. Structural and functional characterization verified the crystalline nature of the nanoparticles and the involvement of plant-derived functional groups in nanoparticle stabilization.

Both the crude plant extracts and their corresponding AgNPs showed clear dose-dependent inhibition of SARS-CoV-2 papain-like protease (PLpro). The AgNP formulations displayed enhanced inhibitory activity compared to the parent extracts, with lower IC_50_ values, indicating improved bioactivity following nanoparticle formation. Among the tested samples, *S. italica*-derived formulations demonstrated comparatively stronger PLpro inhibition. Cytotoxicity evaluation using Vero cells confirmed the high cell viability at concentrations relevant to antiviral activity, supporting the biocompatibility of both extracts and AgNPs.

Altogether, the results confirm that Phyto-formulated silver nanoparticles derived from *S. italica* and *V. reflexa* possess significant antiviral activity against PLpro while maintaining low cytotoxicity. These findings validate the effectiveness of green synthesis using medicinal plant extracts and support the potential application of plant-derived AgNPs as antiviral agents.

## Figures and Tables

**Table 1:** The mass and percentage yield of the dry extracts obtained from *Senna italica* and *Vepris reflexa* leaves.

Extracts	*S. italica* Mass (g)	*S. italica* Yield (%)	*V. reflexa* Mass (g)	*V. reflexa* Yield (%)
Saponins	16.6855	10.04	73.1831	25.96
Flavonoids	29.3898	18.58	52.2389	16.53

Note: The percentage yield is calculated based on the initial plant material weight.

**Table 2:** Particle size, PDI, and seta potential values of the synthesized SFs-AgNPs.

Synthesized SFs-AgNPs	Particle Size (nm)	PDI	Zeta Potential (±mV)
SS-AgNPs	189.9	0.35	+57.9
SF-AgNPs	98.0	0.15	−35.0
VS-AgNPs	35.46	0.11	+41.0
VF-AgNPs	103.3	0.12	+59.5

SS-AgNPs: silver nanoparticles with saponin *S. italica*, SF-AgNPs: silver nanoparticles with flavonoid *S. italica*, VS-AgNPs: silver nanoparticles with saponin *V. reflexa*, VF-AgNPs: silver nanoparticles with flavonoids *V. reflexa*.

**Table 3:** IC_50_ values of plant extracts and synthesized AgNPs against SARS-CoV-2 PLpro enzyme.

Sample	IC_50_ (mg/mL)
SF	~0.06
VF	~0.06
SS	~0.08
VS	~0.07
SS-AgNPs	~0.06
SF-AgNPs	~0.06
VS-AgNPs	~0.07
VF-AgNPs	~0.06

**Table 4:** Cell viability (%) of extracts and AgNPs.

Concentration (mg/mL)	VS-AgNPs (%)	VF-AgNPs (%)	SS-AgNPs (%)	SF-AgNPs (%)
1.0	153.43	173.40	155.27	223.95
0.5	151.29	156.13	152.56	227.78
0.25	156.25	157.65	159.04	231.39
0.125	161.32	155.96	219.74	287.39

**Table 5:** Mean absorbance values and standard deviations for AgNP-treated cells.

Concentration (mg/mL)	VS-AgNPs	VF-AgNPs	SS-AgNPs	SF-AgNPs
1.0	0.104 ± 0.003	0.118 ± 0.002	0.105 ± 0.005	0.104 ± 0.002
0.5	0.103 ± 0.002	0.118 ± 0.002	0.103 ± 0.001	0.106 ± 0.002
0.25	0.106 ± 0.002	0.107 ± 0.002	0.108 ± 0.006	0.108 ± 0.006
0.125	0.109 ± 0.004	0.106 ± 0.005	0.149 ± 0.012	0.134 ± 0.006

Note: Values are presented as mean ± standard deviation (SD).

**Table 6:** UPLC-MS conditions for the separation and analysis of plant SF extracts.

MS conditions		
Detector	Waters Synapt^®^ G2QTOF	
Calibration mass range	50–1200 *m*/*z*	
Capillary voltage	ESI+ 2.8 KV; ESI− 2.4 KV	
Ionization mode	ESI+ and ESI−	
Source temperature	120 °C	
Sampling cone	25 V	
Extraction cone	4.0 V	
Desolvation temperature	350 °C	
Cone gas flow	10.0 L/h	
Desolvation gas flow	600.0 L/h	
Data management	MassLynx™ Version 4.1 UNIFI	
UPLC conditions		
System	Waters Acquity UPLC^®^	
Column	A Kinetex^®^ 1.7 µm EVO C18 100 Å (2.1 mm ID × 100 mm length)	
Injection volume	5 µL	
Column temperature	50 °C	
Sample temperature	8 °C	
Flow rate	0.3 mL/min	
Mobile phase A	Water + 0.1% formic acid	
Mobile phase B	Acetonitrile + 0.1% formic acid	
Gradient		
Time	%A	%B
	97.0	3.0
	97.0	3.0
	0	100.0
	0	100.0
	97.0	3.0
	97.0	3.0

## Data Availability

The original contributions presented in this study are included in the article. Further inquiries can be directed to the corresponding author.

## Abbreviations

The following abbreviations are used in this manuscript:

AgNP	The synthesized silver nanoparticle
DLS	Dynamic light scattering
DMSO	Dimethyl sulfoxide
DTT	Dithiothreitol
FCC	Face-centered cubic
FTIR	Fourier transform infrared spectroscopy
GC-MS	Gas chromatography–mass spectrometry
Mpro	Inhibitory activity of the SAF extracts and synthesized silver nanoparticles against the SARS-CoV-2 main protease
MTT	3-(4,5-dimethylthiazol-2-yl)-2,5-diphenyltetrazolium bromide
PDI	Polydispersity index
PLpro	SARS-CoV-2 papain-like protease
pXRD	Powder X-ray diffraction
SANBI	South African National Biodiversity Institute
SARS-CoV-2	Severe Acute Respiratory Syndrome Coronavirus 2
SF-AgNP	*Senna italica* silver nanoparticle
SFs	*Senna italica* flavonoids
SPR	Surface plasmon resonance
SS	*Senna italica* saponin
SS-AgNPs	*Senna italica* saponin silver nanoparticles
TEM	Transmission electron microscopy
UV-Vis	Ultraviolet visible
VF	*Vepris reflexa* flavonoid
VF-AgNPs	*Vepris reflexa* flavonoid silver nanoparticles
VS	*Vepris reflexa* saponin
VS-AgNPs	*Vepris reflexa* saponin silver nanoparticles
XRD	X-ray diffraction
